# Acute osimertinib exposure induces electrocardiac changes by synchronously inhibiting the currents of cardiac ion channels

**DOI:** 10.3389/fphar.2023.1177003

**Published:** 2023-05-30

**Authors:** Peiwen Li, Xiaohui Tian, Gongxin Wang, Enshe Jiang, Yanming Li, Guoliang Hao

**Affiliations:** ^1^ Department of Cardiology, Huaihe Hospital of Henan University, Kaifeng, China; ^2^ Department of Pharmacy, Huaihe Hospital of Henan University, Kaifeng, China; ^3^ Department of Research, Scope Research Institute of Electrophysiology, Kaifeng, China; ^4^ Institute of Nursing and Health, Henan University, Kaifeng, China

**Keywords:** osimertinib, QT prolongation, cardiac electrophysiology, delayed conduction, ion channel

## Abstract

**Introduction:** As the third generation of epidermal growth factor receptor tyrosine kinase inhibitor (EGFR-TKI), osimertinib has demonstrated more significant cardiotoxicity than previous generations of EGFR-TKIs. Investigating the mechanism of osimertinib cardiotoxicity can provide a reference for a comprehensive understanding of osimertinib-induced cardiotoxicity and the safety of the usage of this drug in clinical practice.

**Methods:** Multichannel electrical mapping with synchronous ECG recording was used to investigate the effects of varying osimertinib concentrations on electrophysiological indicators in isolated Langendorff-perfused hearts of guinea pigs. Additionally, a whole-cell patch clamp was used to detect the impact of osimertinib on the currents of hERG channels transfected into HEK293 cells and the Nav1.5 channel transfected into Chinese hamster ovary cells and acute isolated ventricular myocytes from SD rats.

**Results:** Acute exposure to varying osimertinib concentrations produced prolongation in the PR interval, QT interval, and QRS complex in isolated hearts of guinea pigs. Meanwhile, this exposure could concentration-dependently increase the conduction time in the left atrium, left ventricle, and atrioventricular without affecting the left ventricle conduction velocity. Osimertinib inhibited the hERG channel in a concentration-dependent manner, with an IC_50_ of 2.21 ± 1.29 μM. Osimertinib also inhibited the Nav1.5 channel in a concentration-dependent manner, with IC_50_ values in the absence of inactivation, 20% inactivation, and 50% inactivation of 15.58 ± 0.83 μM, 3.24 ± 0.09 μM, and 2.03 ± 0.57 μM, respectively. Osimertinib slightly inhibited the currents of L-type Ca^2+^ channels in a concentration-dependent manner in acutely isolated rat ventricular myocytes.

**Discussion:** Osimertinib could prolong the QT interval; PR interval; QRS complex; left atrium, left ventricle, and atrioventricular conduction time in isolated guinea pig hearts. Furthermore, osimertinib could block the hERG, Nav1.5, and L-type Ca^2+^ channels in concentration-dependent manners. Therefore, these findings might be the leading cause of the cardiotoxicity effects, such as QT prolongation and decreased left ventricular ejection fraction.

## 1 Introduction

Osimertinib (AZD9291) is a third-generation epidermal growth factor receptor tyrosine kinase inhibitor (EGFR-TKI). It has been used to treat patients with locally advanced or metastatic non-small-cell lung cancer (NSCLC) (Remon and Planchard, 2015; Economopoulou and Mountzios, 2016). Osimertinib overcame the drug resistance of the first-generation EGFR-TKIs and the drug selectivity of the second-generation EGFR-TKIs (Liu et al., 2016b). It was the first approved targeted medicine for adult patients with locally advanced or metastatic NSCLC progressing on or after EGFR TKI therapy and patients with metastatic EGFR-mutant and acquired EGFR T790M mutation (Mok et al., 2017; Soria et al., 2018). Nevertheless, osimertinib-induced cardiotoxicity events increasingly emerged with its wide use in clinical practice. Studies showed that this cardiotoxicity is significantly higher than those in first- and second-generation EGFR-TKIs. In addition, the risk of QT prolongation, heart failure, and atrial fibrillation increased during drug administration (Anand et al., 2019).

Since the improvement of the guideline on the Non-Clinical Evaluation of the Potential for Delayed Ventricular Repolarization (QT interval prolongation) by Human Pharmaceuticals (S7B) by the International Council for Harmonization of Technical Requirements for Pharmaceuticals for Human Use (ICH) (Food and Drug Administration, 2005; Garcia-Elias and Benito, 2018), potential drug-induced proarrhythmic effects, such as cardiac QT prolongation and T-wave abnormality, have gained much attention (Lee et al., 2010; Kloth et al., 2015). An equivocal finding of decreased contractility was reported in dogs and guinea pigs in an *in vivo* cardiac safety pharmacology study (Administration, 2015). However, in addition to QT prolongation in clinical practice (Abriel and Zaklyazminskaya, 2013; Anand et al., 2019; Kunimasa et al., 2020), decreased left ventricular ejection fraction and heart failure caused by osimertinib were commonly reported (Oyakawa et al., 2017; Watanabe et al., 2017; Anand et al., 2019; Kaira et al., 2020). Therefore, while osimertinib-induced cardiac QT prolongation has been a recent focus, the effects of ventricular dysfunction caused by osimertinib were neglected. Studies showed that the occurrence rate of severe cardiovascular toxicity of grade 3 or higher induced by osimertinib reached 4.9% in patients. The primary manifestations of cardiovascular toxicity are decreased left ventricular ejection fraction and heart failure (Kaira et al., 2020). In another AURA3 clinical trial phase 3 study, approximately 4% of patients in the osimertinib group experienced QT prolongation, and another 5% of patients presented with decreased left ventricular ejection fraction (Anand et al., 2019). However, the underlying mechanism of osimertinib-induced cardiotoxic effects remains unknown.

Due to the gaps between the clinical findings and nonclinical drug testing in predicting the development of cardiotoxicity in humans, it is necessary to use cultured cardiomyocytes and isolated perfused hearts to study osimertinib cardiotoxicity. In this study, we explored osimertinib cardiotoxicity on the heart through multichannel electrical mapping and synchronous ECG tests. The cardiotoxic effects of osimertinib include QT interval prolongation and significantly increased left atrium and ventricle conduction times. These may be the important causes of decreased left ventricular ejection fraction and heart failure. In addition, we further applied the whole-cell patch clamp technique to study the cause of cardiotoxic effects through cellular ion channels. This study provided a reference for the safe administration of osimertinib and a more comprehensive safety evaluation of this drug.

## 2 Materials and methods

### 2.1 Experimental animals

SPF (Beijing) Biotechnology Co. Ltd. provided adult male Dunkin–Hartley guinea pigs (200–300 g) and Sprague–Dawley (SD) rats. All animal experiments were approved by the Animal Research Ethics Committee of Scope Research Institute of Electrophysiology and were performed according to the *NIH*’*s Guide for Care and Use of Laboratory Animals*.

### 2.2 Experiment cell

The whole-cell patch clamps experiment used two selected cell lines, including HEK293 cells that stably express the hERG channel and Chinese hamster ovary (CHO) cells that stably express the Nav1.5 channel. The cells were placed in an incubator with 5% CO_2_ at 37°C and cultured in a complete culture medium of 90% DMEM and 10% fetal bovine serum combined with double antibody. The HEK293 cell lines received 250 μg/mL G418, while the CHO cell lines received 500 μg/mL G418. Acutely isolated myocytes from SD rats were prepared according to the procedure described by Han et al. (2022). The cultured cells and acutely isolated myocytes were prepared on cell slides for electrophysiological studies.

### 2.3 Main reagents and solution preparation

Osimertinib (Selleck, Houston United States, Batch No: S7297) was dissolved in dimethyl sulfoxide (DMSO) to prepare solutions of different concentrations. All other chemical reagents were purchased from Sigma unless otherwise noted.

The Krebs–Henseleit (K–H) solution used for the Langendorff heart perfusion included 119 mM NaCl, 25 mM NaHCO_3_, 4 mM KCl, 1 mM MgCl_2_, 1.2 mM KH_2_PO_4_, 1.8 mM CaCl_2_∙2H_2_O, and 10 mM D-glucose. The K–H perfusate was heated to 37°C and oxygenated with 95% O_2_ and 5% CO_2_ before perfusion through the aorta of hearts isolated from guinea pigs.

Retrograde perfusion was performed through the aorta at an 8 mL/min flow rate with oxygenated (95% O_2_, 5% CO_2_) K–H perfusate at 37°C.

The extracellular solution used for the potassium current recording of hERG channels in HEK293 cells included 125 mM NaCl, 5.4 mM KCl, 10 mM HEPES, 0.333 mM NaH_2_PO_4_, 10 mM glucose, 1.8 mM CaCl_2_, and 1 mM MgCl_2_. The pH was adjusted to 7.4 using NaOH. The intracellular solution used for the potassium current recording of hERG channels included 130 mM KCl, 10 mM NaCl, 0.5 mM MgCl_2_, 5 mM Mg-ATP, 0.5 mM EGTA, 10 mM HEPES, and 0.4 mM Tris-GTP). The pH was adjusted to 7.2 using KOH.

The extracellular solution used for the sodium current recording of Nav1.5 channels in CHO cells included 65 mM NaCl, 65 mM TEACl, 10 mM HEPES, 5 mM CsCl, 1 mM MgCl_2_∙6H_2_O, 1 mM CaCl_2_∙2H_2_O, and 12.5 mM D-glucose. The pH was adjusted to 7.4 using NaOH. The intracellular solution used for the sodium current recording of Nav1.5 channels included 130 mM CsCl, 7 mM NaCl, 1 mM MgCl_2_∙6H_2_O, 5 mM HEPES, 5 mM EGTA, 5 mM Mg-ATP, and 0.4 mM Tris-GTP. The pH was adjusted to 7.2 using CsOH.

The extracellular solution used for the L-type Ca^2+^ current recording in acutely isolated myocytes included 25 mM NaCl, 110 mM TEACl, 6 mM HEPES, 1.8 mM CaCl_2_∙2H_2_O, 1 mM MgCl_2_∙6H_2_O, 10 mM D-glucose, and 5 mM Na-HEPES. The pH was adjusted to 7.4 using NaOH. The intracellular solution used for the calcium current recording of L-type Ca^2+^ channels included 120 mM aspartic acid, 120 mM CsOH^**.**^H_2_O, 10 mM CsCl, 10 mM HEPES, 10 mM EGTA, 5 mM Mg-ATP, and 0.4 mM Tris-GTP. The pH was adjusted to 7.2 using CsOH.

### 2.4 *Ex vivo* Langendorff heart perfusion

The guinea pigs were sacrificed after isoflurane anesthesia. Their chests were opened, and the hearts were quickly removed. The isolated hearts were put in the Langendorff perfusion system. Retrograde perfusion was performed through the aorta at an 8 mL/min flow rate with oxygenated (95% O_2_, 5% CO_2_) K–H perfusate at 37°C. Before starting the experimental procedure, the heart was perfused and monitored for 20 min. The experiment did not begin until the heart had stabilized and returned to a normal rhythm. Hearts with abnormal rhythms were excluded from further experiments.

### 2.5 Electrical mapping and synchronous ECG in isolated hearts

The maximum plasma reported concentration (Cmax) of osimertinib in clinical practice is 0.126 μM (Liston and Davis, 2017) under the condition of 80 mg treatment peros (PO). The results of a human pharmacokinetic study in osimertinib suggested a maximum plasma concentration at a steady state (C_ss,max_) of 525 nM under the condition of an 80 mg treatment dosage. Moreover, the maximum and minimum plasma concentration rate is 1.6 (Brown et al., 2017). This study calculated the C_ss,max_ of osimertinib in guinea pigs at Cmax doses based on the human C_ss,max_. A series of concentrations of 0.8 μM, 2.4 μM, 7.2 μM, and 24 μM of osimertinib were chosen for the test in the isolated guinea pig heart. These concentrations were equivalent to 1.5, 4.5, 15, and 45 times the C_ss,max_ in humans.

Guinea pig hearts were excised and retrogradely perfused through the aorta with K–H solution. We used two cascaded multichannel electrical mapping systems (EMS64-USB-1003, MappingLab Ltd., United Kingdom) to record the extracellular potential (ECP). Two 64-channel multi-electrode arrays (MEA) (MappingLab Ltd., United Kingdom) were used to record the left atrium and left ventricle synchronously. The ECP signals were recorded at a sampling frequency of 10 kHz, displayed, and stored on a computer using EMapRecord 5.7.7 software (MappingLab Ltd., United Kingdom). The ECG signals were amplified (MappingLab Ltd., United Kingdom) and continuously recorded using two electrodes on the right atrium and left ventricle, respectively. The stimulating electrode (MappingLab Ltd., United Kingdom) was placed on the apical region of the heart. EMapScope5.8.1 (MappingLab Ltd., United Kingdom) was used for data collection.

### 2.6 Single-cell electrophysiology recording

MultiClamp700A amplifier, 1550A digital–analog converter, and pCLAMP10.6 software (Molecular Devices, U.S.A.) were used for the whole-cell patch clamp recording. Borosilicate glass recording electrodes (resistance, 2–5 MΩ) were made using a glass microelectrode puller (P-97, Sutter, U.S.A.) and filled with an intracellular solution. The slides with cultured cells were placed in the bath. A single cell was selected and adjusted to a clear field of view. Then, the recording electrode was directed to the cell. The whole-cell configurations were established by applying moderate negative pressure to rupture the cell membrane after the electrode contacted the cell membrane surface, and a giga-ohm seal was made. The electrophysiological signal recording was taken under a 6 KHz filter at room temperature (25°C). The stimulation protocol was as follows: to record the hERG current, the cell was clamped at −80 mV, then depolarized from −80 mV to +40 mV for 3,000 ms, followed by repolarization to −40 mV for 2000 ms. To record the hERG activation current, the cell clamps were first clamped at −80 mV, then depolarized from −50 mV to +50 mV in 10-mV steps. Each command voltage lasted for 1,000 ms. Finally, the cell was repolarized to −40 mV for 2000 ms. For the hERG inactivation current recording, the cell was clamped at −80 mV, repolarized to +40 mV, then depolarized from −130 mV to +40 mV with a 10-mV step. Each command voltage was maintained for 3,000 ms. The cell was finally repolarized to +40 mV for 1,000 ms. To record the Nav1.5 current, the cell was first clamped at −120 mV, then depolarized from −120 mV to −15 mV for 100 ms. For a channel inactivation of approximately 20%, the −80 to −90 mV voltage was clamped for 100 ms. For a channel inactivation of approximately 50%, the clamp voltage was held at approximately −70 mV for 100 ms. To record the L-type calcium current in rat ventricular myocytes, the separated cells were placed in a bath, and cells with clear stripes, complete edges, and involuntary contraction under a microscope were selected for use in the experiments. The recording procedure for the L-type calcium currents adopted the CiPA program, which is the most comprehensive worldwide. The protocol is as follows: the cell is first clamped at −80 mV for 100 ms; then the voltage was stepped to −40 mV for 50 ms to inactivate the Na^+^ current; then, the voltage was stepped to 0 mV for 50 ms to record the L-type Ca^2+^ current. The voltage step was then +30 mV for 200 ms; then the cell was given a ramp stimulation by clamping −80 mV for 100 ms to simulate the repolarization of the action potential.

### 2.7 Data analysis

We applied the Van de Water equation to correct the QT interval. The corrected QT (QTc) by the HR was expressed as QTc = QT − 0.087 × (RR − 1000) (Van de Water et al., 1989). This corrected equation demonstrated a robust positive correlation between the QT interval and cardiac repolarization (Morissette et al., 2015).

Origin 2021 (OriginLab, United States) and GraphPad Prism 9.5 (GraphPad, United States) software were used to plot graphs and analyze data in this study. All experimental data were expressed as means ± SEM. The logistic regression equation was adopted for non-linear concentration–response curve fitting. IC_50_ represents the 50% inhibitory concentration. A repeated-measure one-way ANOVA with Tukey’s multiple comparisons test was used to analyze the data. The rejection criterion for all statistical tests was set at the conventional value of 0.05.

## 3 Results

### 3.1 Effects of varying osimertinib concentrations on the ECG in hearts isolated from guinea pigs

**FIGURE 1:**
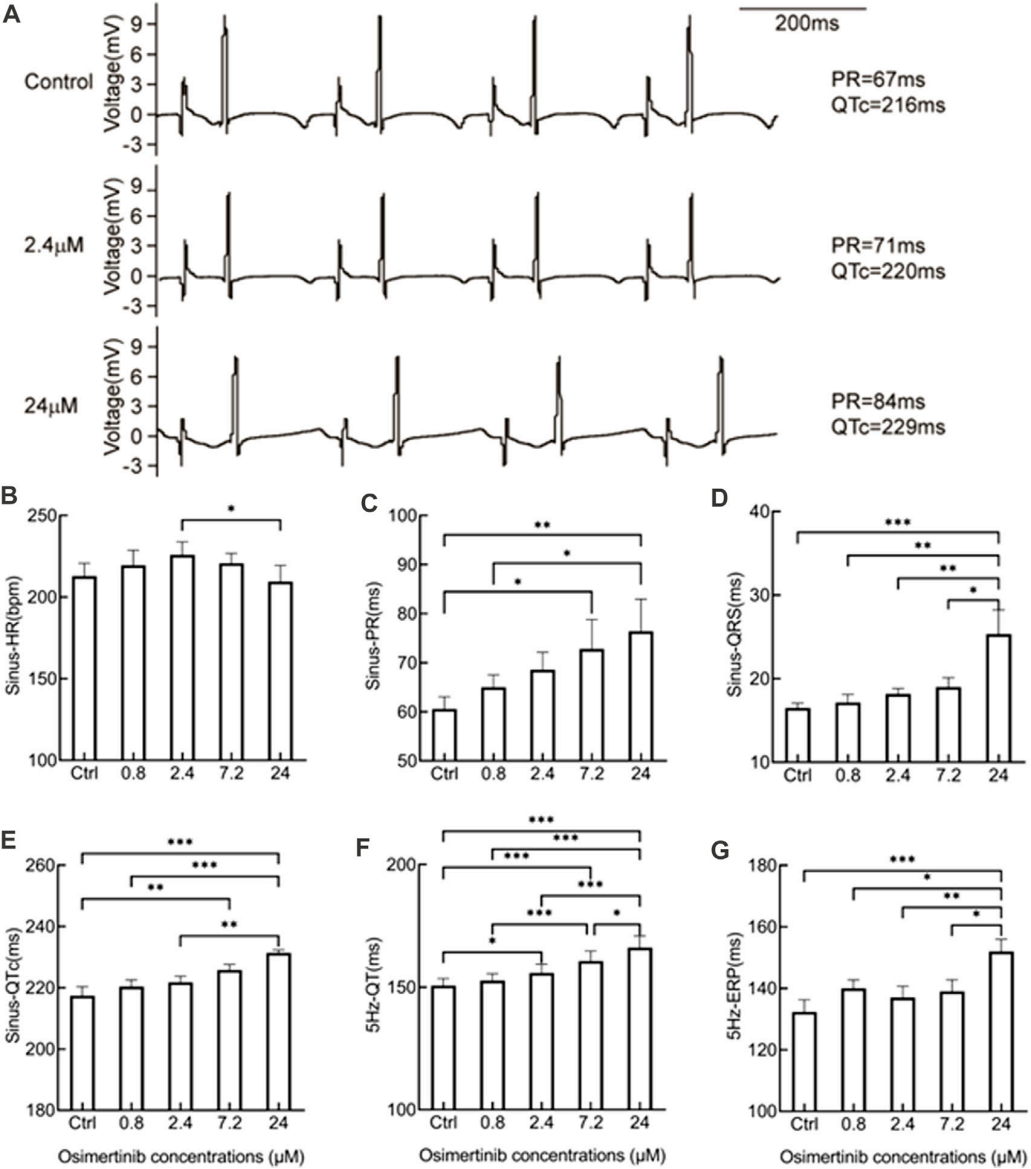
Effects of varying osimertinib concentrations on ECG recordings from normal hearts isolated from guinea pigs. **(A)** Three representative ECG recordings from the isolated hearts of guinea pigs in the control, 2.4 μM osimertinib, and 24 μM osimertinib groups. The bar graph shows the summary data of the HR **(B)**, PR **(C)**, QRS complex **(D)**, and QTc **(E)** under sinus rhythm as well as the QT **(F)** and ERP **(G)** under 5 Hz stimulation in response to the varying osimertinib concentrations. HR, heart rate; PR, interval of P- and R-wave in the ECG recording; QRS, width of the QRS complex wave; QTc, corrected QT interval by HR; QT, interval of Q- and T-wave in the ECG recording; ERP, effective refractory period; *p < 0.05; **p < 0.01; ***p < 0.001.

The QT interval is known to be prolonged with osimertinib use in the clinical setting. In this study, the effects of the acute perfusion of different concentrations of osimertinib on the ECG were evaluated by ECG recordings from hearts isolated from guinea pigs. Figure 1A provides representative traces from one sample treated with three different concentrations of osimertinib. As supported statistically in the following sentences, the PR and QT interval at the sinus rhythm were increased following treatment with 2.4 or 24 μM of osimertinib compared to the control. The osimertinib treatment also made T-wave abnormal changes in the ECG profile. Repeated-measures one-way ANOVA showed that except for the HR (*F*_(4,20)_ = 3.071, *p* = 0.040, n = 6, Figure 1B), which was induced by treatment with 2.4 μM of osimertinib, which was significantly increased compared to 24 μM of osimertinib (*p* = 0.039). The osimertinib concentration had a significant effect on the ECG electrical indicators, including the PR interval (*F*_(4,16)_ = 7.752, *p* = 0.0011, n = 5, Figure 1C), the width of the QRS complex (*F*_(4,20)_ = 7.600, *p* = 0.0007, n = 6, Figure 1D), the QTc (QT interval with heart rate correction, *F*_(4,16)_ = 12.73, *p* < 0.001, n = 5, Figure 1E) at sinus rhythm, as well as the QT at 5 Hz forced pacing rhythm (*F*_(4,20)_ = 29.16, *p* < 0.001, n = 6, Figure 1F), in which the HR was captured at a fixed 5 Hz frequency by apical stimulation. Of note is that the highest osimertinib concentration (24 μM) produced a relatively significant prolongation of the PR interval, QRS, QTc, and 5 Hz-QT. This study also used the S1S2 stimulation procedure to measure the ventricular effective refractory period (ERP). The results showed that the osimertinib concentration had significant effects on the ERP (*F*_(4,20)_ = 7.291, *p* < 0.001, n = 6, Figure 1G) with significantly increased ventricular ERP by 19.67 ± 5.35 ms (*p* < 0.001) for the 24 μM osimertinib treatment (Figure 1G) compared with the control.

### 3.2 Effects of varying osimertinib concentrations on conduction times in hearts isolated from normal guinea pigs

**FIGURE 2:**
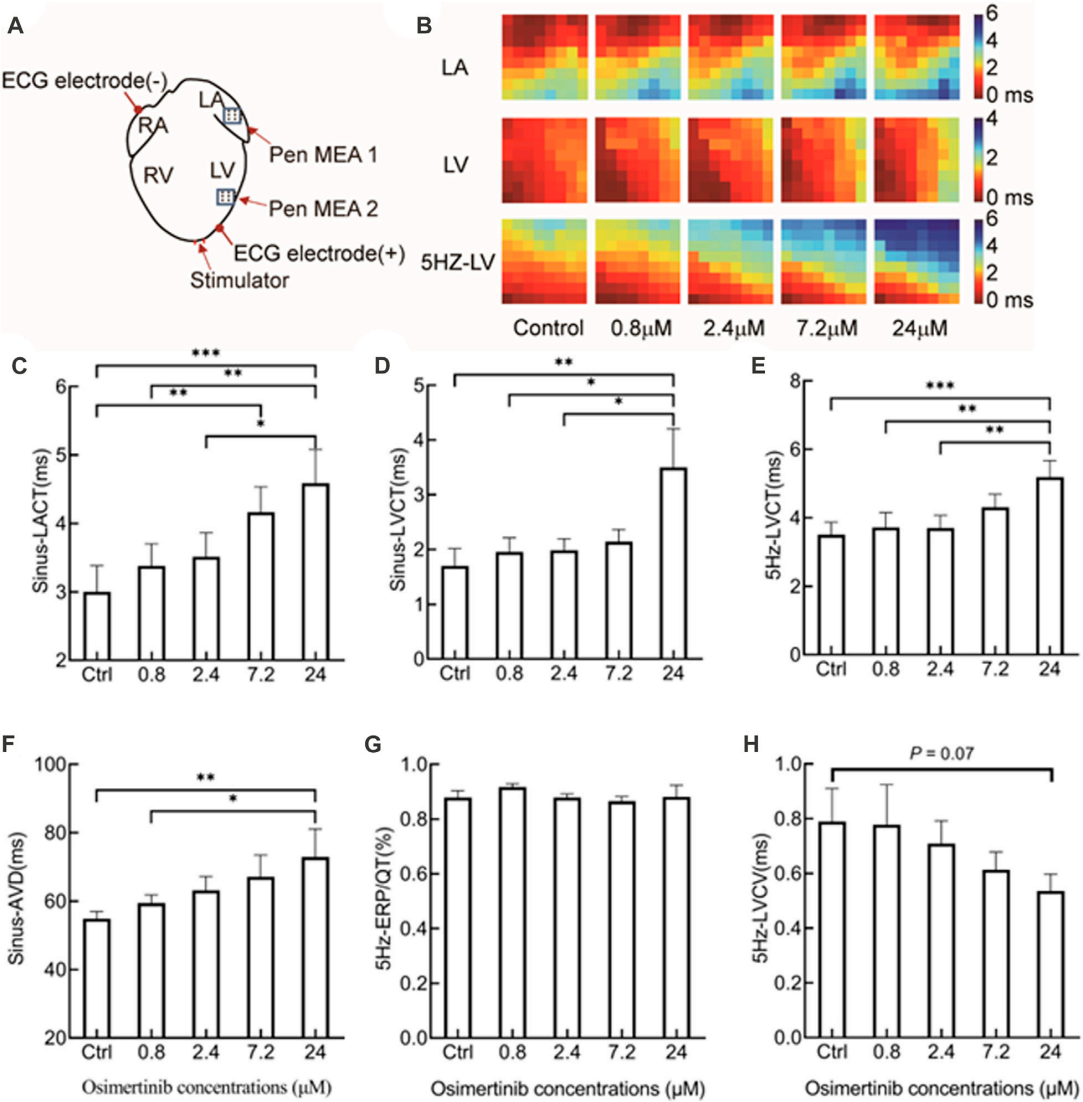
Effects of varying osimertinib concentrations on conduction profiles of hearts isolated from guinea pigs by electrical mapping technique. **(A)** Position sites of the electrodes for the apical stimulation, multi-electrode assay (MEA), and ECG in the electrical mapping test. **(B)** Representative isochronograms of the conduction time of the left atrium and left ventricular under sinus rhythm, and from the left ventricle under 5 Hz stimulation. The bar graph shows summary data of sinus-LACT **(C)**, sinus-LVCT **(D)**, 5 Hz-LVCT **(E)**, sinus-AVD **(F)**, 5 Hz-ERP/QT **(G)**, and 5 Hz-LVCV **(H)** responses to the varying osimertinib concentrations. Sinus-LACT, left atrium conduction time under sinus rhythm; sinus-LVCT, left ventricle conduction time under sinus rhythm; 5-Hz-LVCT, left ventricle conduction time under 5 Hz stimulation; sinus-AVD, atrioventricular delay under sinus rhythm; 5-Hz-LVCV, left ventricle conduction velocity under 5 Hz stimulation; *p < 0.05; **p < 0.01; ***p < 0.001.

The position sites of stimulating, multichannel MEA, and ECG electrodes in electrical mapping experiments are shown in Figure 2A. The epicardial isochronous activation mapping graph during sinus rhythm or forced pacing induced by 5 Hz stimulation was depicted as the time difference between the earliest activated site and a single activated site on each channel. In addition, the activation time was calculated as the maximum negative slope of the activation waveform.

After the administration of varying concentrations of osimertinib, the conduction times of the left atrium (LA), left ventricular (LV), and left ventricular under 5 Hz stimulation (5 Hz-LV) were measured by multichannel electrical mapping. Figure 2B shows representative isochronous electrical conduction maps, in which the conduction time of the heart was gradually prolonged with increasing drug concentration. Compared with the control, osimertinib prolonged the conduction time of the LA (*F*_(4,12)_ = 11.08, *p* < 0.001, n = 4, Figure 2C), LV (*F*_(4,20)_ = 4.752, *p* = 0.007, n = 6, Figure 2D), and 5 Hz-LV (*F*_(4,20)_ = 7.888, *p* < 0.001, n = 6, Figure 2E), respectively, in a concentration-dependent manner. The prolongation effects of osimertinib on the conduction time of LA, LV, and 5 Hz-LV reached a maximum at the highest osimertinib concentration (24 μM).

In this study, the difference between the left atrium and left ventricular activation time after osimertinib treatment was used to represent the atrioventricular delay at sinus rhythm (sinus-AVD). We found that the varying osimertinib concentrations also showed prolonged sinus-AVD (*F*_(4,16)_ = 5.517, *p* = 0.005, n = 5, Figure 2F). However, the varying osimertinib concentrations had no effects on the 5 Hz-ERP/QT (*F*_(4,20)_ = 0.6397, *p* = 0.640, n = 6, Figure 2G). In addition, the conduction velocities of the left ventricular under 5 Hz stimulation (5 Hz-LVCV) in response to varying osimertinib concentrations showed a descending trend, although the effects of varying osimertinib concentrations on 5 Hz-LVCV did not reach statistical difference (*F*_(4,20)_ = 2.875, *p* = 0.051, n = 6, Figure 2H).

### 3.3 Osimertinib inhibition of the currents of hERG and Nav1.5 channels

**FIGURE 3:**
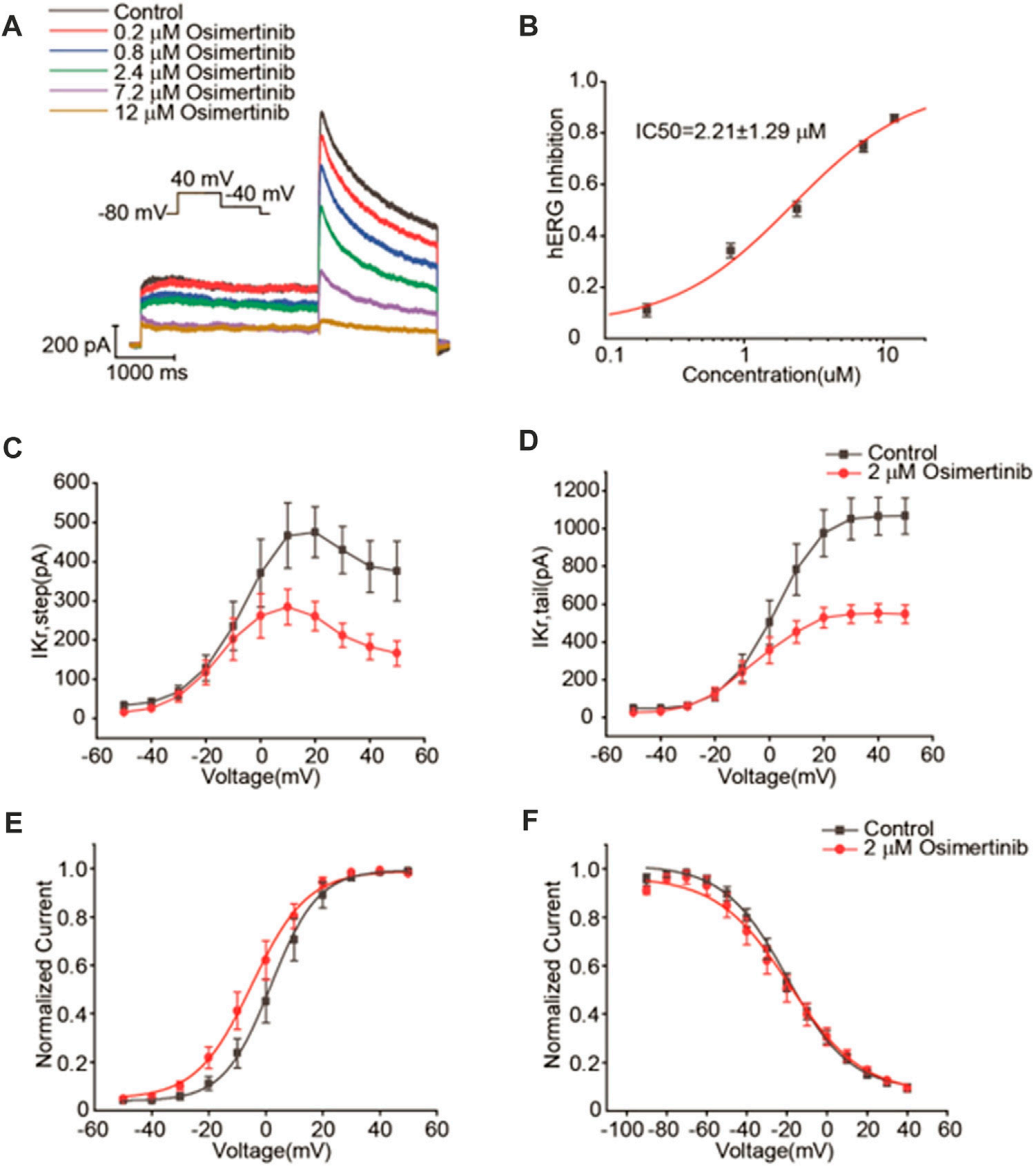
Inhibitory effects of osimertinib on hERG channels. **(A)** Representative traces of the Ikr currents in response to varying concentrations of osimertinib. **(B)** Dose–response curve of osimertinib for the hERG channel. **(C)** Current–voltage curve of the hERG channel activation current. **(D)** Current–voltage curve of the tail current of the hERG channel. **(E)** Activation curve of the hERG channel after fitting with the Boltzmann equation. **(F)** Inactivation curve of the hERG channel after fitting with the Boltzmann equation.

The drug-induced prolongation of ventricular action potential duration (APD) and QT interval is often associated with inhibited repolarization of I_kr,_ the rapid component of the cardiac delayed rectifier potassium current (Liu et al., 2016a). In contrast, the slowdown of the conduction velocity of the action potential is often associated with the inhibition of the current of Nav1.5 channels. For example, all type 1 antiarrhythmic drugs are related to a slowed action potential conduction. Therefore, this study focused on the effects of osimertinib on the currents of hERG and Nav1.5 channels. In this study, the change in the potassium current of hERG channels was recorded by using the whole-cell patch-clamp technique. Varying concentrations of osimertinib (0 μM, 0.2 μM, 0.8 μM, 2.4 μM, 7.2 μM, and 12 μM) were applied to HEK293 cells transfected with hERG channels in sequence. The results showed that osimertinib could inhibit the currents of hERG channels in a concentration-dependent manner. The currents of hERG channels were almost completely inhibited at a concentration of 12 μM (Figure 3A). The dose–response curve could be obtained by fitting the concentration–response relationship using a logistic regression equation (Figure 3B, n = 9). The results showed that the 50% inhibitory concentration (IC_50_) of osimertinib on the current of hERG channels was 2.21 ± 1.29 μM.

According to the IC_50_ of osimertinib inhibition of the currents of hERG channels (2.21 μM), 2 μM osimertinib was selected for the channel kinetics study. From the current–voltage (I–V) curves for hERG potassium activation using the means of the activated terminal currents, 2 μM osimertinib significantly inhibited the currents of hERG channels (Figure 3C). The tail currents also demonstrated the same results (Figure 3D). The Boltzmann equation was used to fit the activation curve after tail current normalization, and the midpoint voltage (V_1/2_) could be obtained from the activation curve. The V_1/2_ of the 2 μM osimertinib activation curve was negatively shifted by 6.68 mV (1.81 ± 0.56 mV vs. −4.87 ± 3.13 mV, n = 6, *p* < 0.05); that is, the hERG channel activation curve shifted to the left after the administration of 2 μM of osimertinib (Figure 3E). After tail current normalization, the inactivation curves were fitted with the Boltzmann equation with no significant change in V_1/2_ (*p >* 0.05, n = 9) (Figure 3F). This finding indicated no substantial change in hERG potassium channel inactivation after the administration of 2 μM of osimertinib.

**FIGURE 4:**
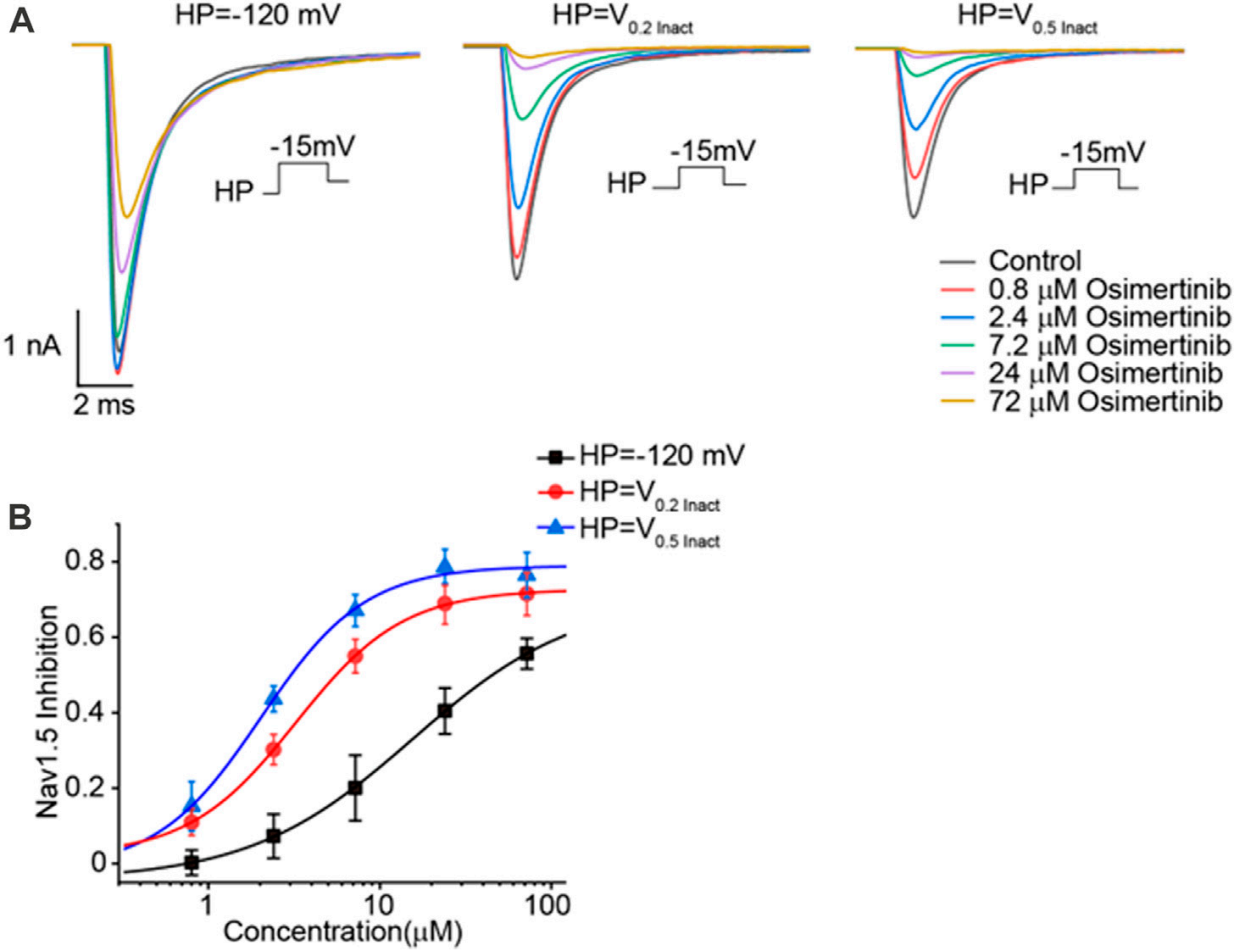
Inhibitory effects of osimertinib on the Nav1.5 channel. **(A)** Inhibitory effects of varying osimertinib concentrations on Nav1.5 channels at different HP levels. **(B)** Dose–response curve of osimertinib on Nav1.5 channels at different HPs. HP, holding potential; Inact, inactivation.

The currents of Nav1.5 channels were recorded by voltage-clamp mode with the whole-cell configuration in CHO cells transfected with Nav1.5 channels. Varying osimertinib concentrations (0 μM, 0.8 μM, 2.4 μM, 7.2 μM, 24 μM, and 72 μM) were applied to the chamber containing CHO cells. A three-stage voltage clamp (no inactivation, 20% inactivation, and 50% inactivation) was used to record the sodium current of the Nav1.5 channels. The results showed that the osimertinib could inhibit the sodium current in a concentration-dependent manner, with especially inhibitory effects on Nav1.5 channels, which was also inactivation-dependent. The sodium currents of Nav1.5 channels were almost completely inhibited at the highest osimertinib concentration (72 μM) under 20% and 50% inactivation (Figure 4A). The dose–response curve of osimertinib inhibition of the Nav1.5 channel was obtained by fitting the concentration–response relationship using a logistic regression equation (Figure 4B). Furthermore, the inhibitory effect of osimertinib on Nav1.5 channels gradually increased. The inhibition effect was more significant when the drug concentration was at its highest. The IC_50_ values under the no inactivation, 20% inactivation, and 50% inactivation conditions were 15.58 ± 0.83 μM (n = 8), 3.24 ± 0.09 μM (n = 8), and 2.03 ± 0.57 μM (n = 8), respectively.

**FIGURE 5:**
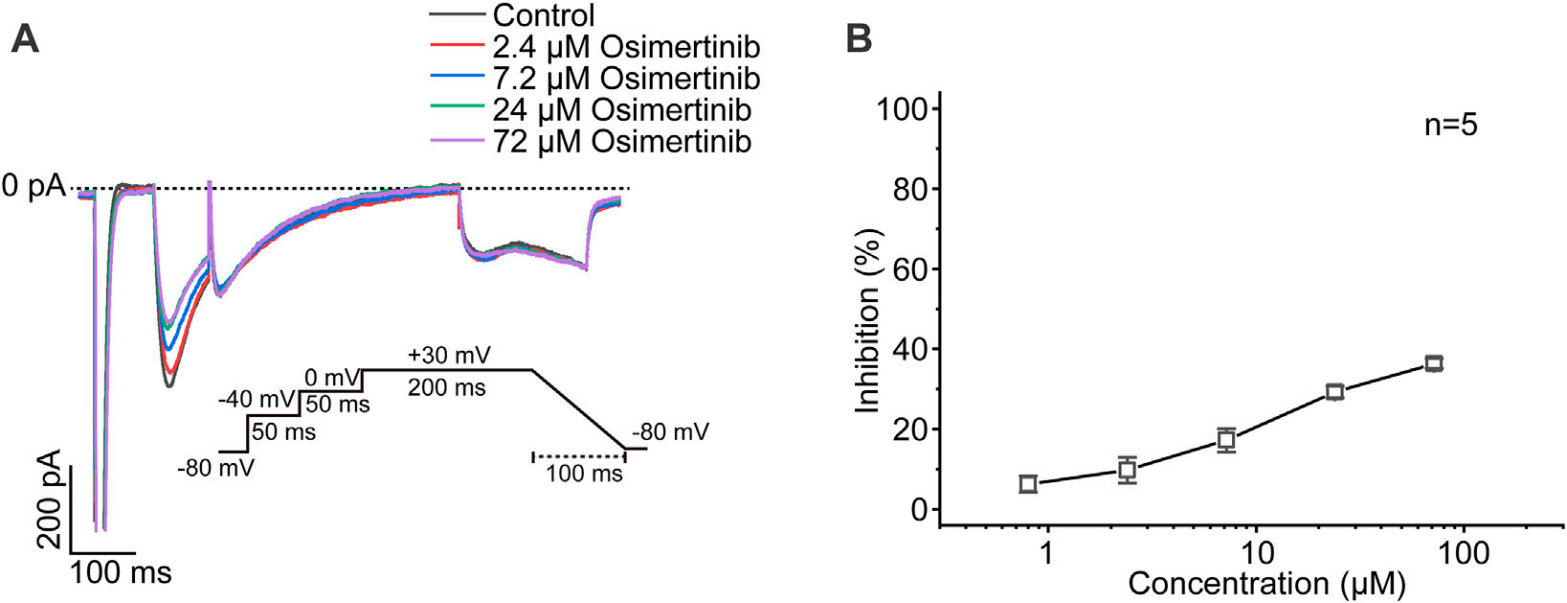
Inhibitory effects of osimertinib on L-type Ca^2+^ channels. **(A)** Representative figure showing the effects of varying osimertinib concentrations on the currents of L-type Ca^2+^ channels. **(B)** Dose–response curve for the inhibition of varying osimertinib concentrations on the currents of L-type Ca^2+^ channels.

The currents of L-type Ca^2+^ channels were recorded from the acutely isolated ventricular myocyte from SD rats by voltage-clamp mode with the whole-cell configuration. Varying concentrations of osimertinib (0 μM, 0.8 μM, 2.4 μM, 7.2 μM, 24 μM, and 72 μM) were applied to the chamber containing ventricular myocytes. A representative current of L-type Ca^2+^ channels induced by varying osimertinib concentrations is shown in Figure 5A. The results showed that osimertinib slightly inhibited the calcium current in a concentration-dependent manner (Figure 5B). For example, 2.4 μM of osimertinib had a 9.77% inhibition rate on the current of L-type calcium channels.

## 4 Discussion

In this study, we investigated the effects of osimertinib on cardiac electrophysiological activity and ion channel activity using hearts isolated from guinea pigs, HEK293 cells transfected with the hERG channel, and CHO cells transfected with the Nav1.5 channel. We proved that osimertinib could prolong the PR interval, QT interval, and QRS complex and cause abnormal changes in the T-wave. In addition, osimertinib also slowed the conduction of the left atrium and left ventricle in the hearts isolated from guinea pigs. This result was further supported in the ion channel study, which demonstrated that osimertinib could effectively inhibit hERG and Nav1.5 channels and slightly inhibit L-type Ca^2+^ channels. Furthermore, the QT prolongation was associated with the inhibition of hERG potassium channels; this slowed conduction was consistent with the inhibition of Nav1.5 sodium channels. These different levels of recordings strengthened the validity and reproducibility of the data, which are essential for drug development.

A human pharmacokinetic study reported that the maximum plasma concentration (Cmax) of osimertinib (brand name: Tagrisso) at a dose of 80 mg by the administration route of PO in clinical practice was 0.126 μM. The FDA label for osimertinib reported a mean volume of distribution at a steady state of osimertinib of 918 L and a plasma protein binding of osimertinib of 95% (FDA, 2020). The Cmax value of the osimertinib in the present study represented the peak exposure observed at the highest clinically recommended dose delivered as a single PO administration in the clinic. Most anticancer drugs are provided by repeated administration, which may lead to accumulation. Therefore, some anticancer drugs may achieve higher exposures at a steady state. The reported C_ss,max_ of osimertinib at the 80 mg approved dose was 525 nM in the pharmacokinetics of a population of patients with non-small cell lung cancer administered osimertinib (Brown et al., 2017). The administration of osimertinib orally once daily resulted in an approximately three-fold accumulation with steady-state exposures after 15 days of dosing. The median time to the Cmax of osimertinib was 6 h and the mean volume of distribution at a steady state of osimertinib was 918 L (Administration, 2020). Considering its adsorption to cells and/or perfusion apparatus due to its high distribution volume, the drug accumulation in tissue and cells may be exposed to concentrations higher than those measured in plasma. In general, the drug concentrations used in *in vitro* studies were far greater than those that could be realistically achieved in a clinical setting (Smith and Houghton, 2013). In this cardiac electrophysiological study, we used a series of osimertinib concentrations (0.8 μM, 2.4 μM, 7.2 μM, and 24 μM), which were equivalent to the 1.5-, 4.5-, 15-, and 45-fold of the C_ss,max_ of osimertinib concentration in clinical practice. We used this concentration series to investigate the drug’s effects on the ECG and cardiac conductivity in hearts isolated from guinea pigs, as well as cells transfected with sodium and potassium channels.

The latest product monograph for osimertinib recommends dose modification for cardiac toxicities in the following conditions: ①osimertinib should be permanently discontinued when symptomatic congestive heart failure or QTc interval prolongation with signs/symptoms of serious arrhythmia are detected; ②osimertinib should be withheld until the QTc interval is <481 msec or recovery to baseline if the baseline QTc is ≥481 msec, then restart at a reduced dose (40 mg) when the QTc interval is >500 msec on at least two separate ECGs (Inc, 2023). QTc prolongation may lead to an increased risk of ventricular arrhythmias including torsade de pointes. The prolongation effect of a drug on ventricular APD and QTc interval is often associated with the rapidly activating delayed rectifier potassium current (I_Kr_) (Kannankeril et al., 2010; Liu et al., 2016a). Previous studies showed that the free or unbound plasm Cmax of one drug is considered safe when the free Cmax of the drug is one-thirtieth of the concentration at which the drug can block the I_kr_ (Redfern et al., 2003). The closer the drug concentration of the blocking I_Kr_ current to the Cmax plasma concentration of the drug, the higher the risk of the drug causing severe ventricular arrhythmias and sudden death (De Bruin et al., 2005). Our results demonstrated that osimertinib inhibited hERG potassium currents in a concentration-dependent manner. From this study, we obtained the IC_50_ value (2.21 μM) for osimertinib inhibition of the currents of hERG channels transfected into HEK293 cells. Osimertinib inhibited the hERG potassium channel in CHO cells, with an IC_50_ of 0.69 μM (Inc, 2023). In another *in vitro* Ion Works Quattro study, Zhang et al. evaluated the inhibition of the hERG channel by osimertinib in HEK293 cells and obtained the IC_50_ value 0.57 μM (Zhang et al., 2017; Jin et al., 2018). The relatively large difference in the IC_50_ values for osimertinib inhibition of hERG channels may be attributed to differences in cells, temperature settings, or methodology used in the respective experiments. In our study of isolated whole hearts using electrical mapping, we found that the effects of 2.4 μM of osimertinib on the sinus-QTc interval were not significant, although 2.4 μM of osimertinib had a prolonging effect on the 5 Hz-QT interval, as showed in Figures 1E, F. This may be attributed to our findings from the study on the hERG potassium channel. The 2.0 μM osimertinib concentration could trigger an early activation of the hERG potassium channel without having a significant effect on the inactivation of this channel. This may be one reason why the QT interval prolongation induced by 2.4 μM of osimertinib was not significant, although 2.0 μM of osimertinib (close to the IC_50_ value of 2.21 μM) showed potent inhibition of hERG channels. A clinical study of osimertinib in a Phase II trial (OCEAN) reported a plasma osimertinib Cmax of 1270 nM in patients administered oral osimertinib at a dose of 80 mg once daily (FDA, 2020). Considering the 95% protein binding rate of osimertinib in the plasma, the 30-fold increase in free plasm concentration is close to the IC_50_ of osimertinib inhibition of hERG channels in our study. Therefore, a dose of 80 mg once daily still has the potential risk to cause arrhythmias in clinical practice.

The osimertinib-induced QT interval prolongation in guinea pig hearts is not necessarily caused by the inhibition or activation of a single channel. This finding might be a combined result of multichannel inhibitions. Therefore, the risk evaluation of the drug-induced arrhythmia and sudden death could depend not only on its anti-hERG activity. In addition, high concentrations of osimertinib can prolong ventricular ERP. ERP prolongation has been shown to lead to antiarrhythmic. Therefore, the smaller the ERP value, the greater the risk of arrhythmia (Hondeghem, 2008). The prolongation of ventricular ERP by osimertinib has a specific antiarrhythmic effect. This may also be one explanation for why osimertinib can lead to arrhythmic and heart failure events, but the relative rarity of these events in clinical practice. Another matter requiring attention when explaining the osimertinib cardiotoxicity of QT prolongation and heart failure in the clinic from this study is the osimertinib Cmax, protein binding, and the time to onset of osimertinib in patients. Liston and Davis (2017) reported a clinical Cmax of osimertinib of approximately 0.126 μM and an osimertinib protein binding ratio of 99%. The median (IQR) timing of the appearance of osimertinib cardiotoxicity in clinical practice was 103 [72–306] days. In our study, the 2.4 μM dose of osimertinib showed effects on the 5 Hz-QT interval but not on the sinus-QTc interval. This concentration is approximately 20-fold higher than the Cmax for osimertinib and 4 times the C_ss,max_ in clinical practice. In addition, due to the very high protein binding ratio in the plasma and the very long interval between the start of treatment and the appearance of cardiotoxicity, the data from our acute non-clinical study are insufficient to explain the clinical outcomes in the osimertinib application. This may be one explanation for why QT prolongation seldom occurs in the clinical setting in patients administered a treatment dose of 80 mg.

During treatment of NSCLC with osimertinib in patients, decreased left ventricular ejection fraction often occurs, although QT interval prolongation also exists. However, the potential mechanism of this osimertinib-induced cardiotoxicity remains unknown. Generally, the status of the Nav1.5 channel is associated with cardiac electrical conduction and action potential depolarization (Song and Shou, 2012) and affects heart function. The results from the isolated hearts and patch-clamp experiment showed slowed depolarization and atrioventricular block at high concentrations of osimertinib. Osimertinib inhibited Nav1.5 channel current in a concentration-dependent, inactivation-independent manner. The IC_50_ of osimertinib inhibition of the Nav1.5 channel at non-inactivation was 15.58 μM, which is approximately 30 times the Cmax (525 nM). The IC_50_ values of osimertinib at 20% and 50% channel inactivation (3.24 μM and 2.03 μM) were 4–6 times the Cmax. The reported IC_50_ of osimertinib inhibition of the human Nav1.5 (hNav1.5) was >33 μM, and the inhibition rate of osimertinib blocking hNav1.5 was 29% at 33 μM (Administration, 2015). When the channel inactivation is at 20%, the clamp voltage between −80 and −90 mV is close to the resting potential of human cardiomyocytes. These results are in line with the findings from hearts isolated from normal guinea pigs; namely, that osimertinib increased the cardiac conduction time, PR interval QRS duration, ERP, and sinus-AVD in a concentration-dependent manner. Osimertinib increased in the QT interval in the conscious telemetry of dogs (n = 4) following the oral administration of a single dose of 60 mg/kg, which produced a mean osimertinib Cmax value of 2.51 μM (Inc, 2023). Therefore, considering its IC_50_ and the inhibition of the L-type Ca^2+^ channel, osimertinib may have significant effects on the QRS duration and PR interval due to the accumulation of osimertinib in tissue after chronic administration in clinical practice, as osimertinib accumulation in tissue means a higher drug exposure than the plasma concentration, which would impact the exposure-related drug safety assessment. Moreover, the higher the clamp voltage, the stronger the blocking effect of osimertinib on Nav1.5 and L-type Ca^2+^ channels.

The resting potential of the atrium is often higher than that of the ventricle; thus, the atrium is more subjected to drug effects. This might be one explanation for the adverse reactions to the drug, including atrial fibrillation in the clinical administration of osimertinib. In addition, the slowing of conduction and depolarization affects the heterogeneity of systole. The blocking effect of osimertinib was gradually aggravated with increasing concentration. A high degree of atrioventricular block also occurred at high osimertinib concentrations. Conduction blockage is one risk factor for osimertinib-induced arrhythmic events. The worsening of this conduction blockage also affects ventricular contraction. This may be one explanation for the decreased left ventricular ejection fraction, and heart failure in patients with NSCLC in clinical practice. Across clinical trials, 3.0% of the 1,479 osimertinib-treated patients reported cardiomyopathy events including heart failure and decreased ejection fraction (Administration, 2020). However, the left ventricle dysfunction and heart failure observed in clinical practice are more likely attributed to structural damage of the myocardia rather than functional inhibition of cardiac ion channels, since contractile dysfunction in patients has a slow onset, occurring a minimum of 2 weeks after treatment. Osimertinib and its active metabolites also inhibit wild-type EGFR as well as HER2, HER4, ACK1, and BLK at clinically relevant concentrations. Moreover, osimertinib also inhibited downstream signaling in HER2- and HER3-overexpressing cell lines (Administration, 2015). A recent study showed that osimertinib caused myocardial death *via* inhibition of the pro-survival ERG signaling pathway (Singh et al., 2022). Therefore, cardiac monitoring including ECG and LVEF assessment at baseline and during osimertinib treatment should be provided in clinical practice in patients with cardiac risk factors.

In addition, we found that the HR increased when applying osimertinib at a low concentration of 2.4 μM under the sinus rhythm, although it did not reach a significant level when compared to the control. The HR decreased with gradually increasing osimertinib concentration, with a significant difference in HR between 2.4 μM and 24 μM osimertinib. However, the HR changes did not differ significantly between the control and the high (24 μM) concentration of osimertinib. This was probably associated with the decreased autorhythmicity caused by the inhibition by osimertinib of Nav1.5 sodium channels and L-type Ca^2+^ channels in this study.

In conclusion, our findings suggested that only blocking the hERG potassium channel with 2 μM osimertinib did not lead to osimertinib-induced QT prolongation. Osimertinib showed substantial cardiovascular risks when it simultaneously blocked the hERG, Nav1.5, and L-type Ca^2+^ channels. These effects are likely to lead to cardiovascular adverse events such as decreased left ventricular ejection fraction, heart failure, QT prolongation, and atrial fibrillation in some patients with NSCLC. These cardiovascular issues will significantly lower patients’ quality of life and increase the occurrence and mortality in high-risk populations if osimertinib is not managed correctly. Furthermore, patients with NSCLC with related underlying diseases should be more cautious in choosing osimertinib treatment. Frequent ECG monitoring should be performed while using this drug. Patients with NSCLC must take these changes seriously regarding the cardiac safety of osimertinib use.

## Data Availability

The original contributions presented in the study are included in the article/Supplementary Material, further inquiries can be directed to the corresponding author.
