# Corrigendum: Acute osimertinib exposure induces electrocardiac changes by synchronously inhibiting the currents of cardiac ion channels

**DOI:** 10.3389/fphar.2023.1242042

**Published:** 2023-06-23

**Authors:** Peiwen Li, Xiaohui Tian, Gongxin Wang, Enshe Jiang, Yanming Li, Guoliang Hao

**Affiliations:** ^1^ Department of Cardiology, Huaihe Hospital of Henan University, Kaifeng, China; ^2^ Department of Pharmacy, Huaihe Hospital of Henan University, Kaifeng, China; ^3^ Department of Research, Scope Research Institute of Electrophysiology, Kaifeng, China; ^4^ Institute of Nursing and Health, Henan University, Kaifeng, China

**Keywords:** osimertinib, QT prolongation, cardiac electrophysiology, delayed conduction, ion channel

In the published article, there was an error in **affiliation** 1. Instead of “School of Clinical Medicine, Henan University, Kaifeng, China”, it should be “Department of Cardiology, Huaihe Hospital of Henan University, Kaifeng, China”.

The authors apologize for this error and state that this does not change the scientific conclusions of the article in any way. The original article has been updated.

